# Engineered Jurkat Cells for Targeting Prostate-Specific Membrane Antigen on Prostate Cancer Cells by Nanobody-Based Chimeric Antigen Receptor

**DOI:** 10.29252/ibj.24.2.81

**Published:** 2019-10-23

**Authors:** Mahmoud Hassani, Fatemeh Hajari Taheri, Zahra Sharifzadeh, Arash Arashkia, Jamshid Hadjati, Wytske M. van Weerden, Shahriyar Abdoli, Mohammad Hossein Modarressi, Mohsen Abolhassani

**Affiliations:** 1 Department of Molecular Medicine, School of Advanced Technologies in Medicine, Tehran University of Medical Sciences, Tehran, Iran;; 2 Department of Immunology, Hybridoma Lab, Pasteur Institute of Iran, Tehran, Iran;; 3 Department of Virology, Pasteur Institute of Iran, Tehran, Iran;; 4 Department of Immunology, School of Medicine, Tehran University of Medical Sciences, Tehran, Iran;; 5 Department of Urology, Erasmus Medical Center, Rotterdam, Netherlands

**Keywords:** Chimeric antigen receptor, Immunotherapy, Prostate cancer, PSMA

## Abstract

**Background:**

Recently, modification of T cells with CAR has been an attractive approach for adoptive immunotherapy of cancers. Typically, CARs contain a scFv. Most often, scfvs are derived from a monoclonal antibody of murine origin and may be a trigger for host immune system that leads to the T-cell clearance. Nanobody is a specific antigen-binding fragment derived from camelid that has great homology to human VH and low immunogenic potential. Therefore, in this study, nanobody was employed instead of scFv in CAR construct.

**Methods:**

In this study, a CAR was constructed based on a nanobody against PSMA (NBPII-CAR). At first, Jurkat cells were electroporated with NBPII-CAR, and then flow cytometry was performed for NBPII-CAR expression. For functional analysis, CAR T cells were co-cultured with prostate cancer cells and analyzed for IL-2 secretion, CD25 expression, and cell proliferation.

**Results:**

Flow cytometry results confirmed the expression of NBPII-CAR on the transfected Jurkat cells. Our data showed the specificity of engineered Jurkat cells against prostate cancer cells by not only increasing the IL-2 cytokine (about 370 pg/ml) but also expressing the T-cell activation marker CD25 (about 30%). In addition, proliferation of engineered Jurkat cells increased nearly 60% when co-cultured with LNCaP (PSMA^+^), as compared with DU145 (PSMA^-^).

**Conclusion:**

Here, we describe the ability of nanobody-based CAR to recognize PSMA that leads to the activation of Jurkat cells. This construct might be used as a promising candidate for clinical applications in prostate cancer therapy.

## INTRODUCTION

Prostate cancer is the second most common cancers among men in industrialized countries, and it is the third leading cause of cancer death in men^[^^1^^]^. Although all localized prostate cancers can effectively be treated and cured by conventional therapeutic approaches such as surgery or radiation therapy, therapeutic options are limited for metastases or hormone-refractory disease. Thus, new approaches are needed to treat metastatic prostate cancers^[^^2^^].^

Recently, adoptive immunotherapy by using T cells engineered with CARs has had an acceptable potential for the treatment of metastatic cancers. This molecular building is constructed by attaching an antigen recognition domain of antibodies to co-stimulatory and the zeta (ζ) signaling domains of T-cell receptor. CARs can redirect and activate T cells to the targeted tumor cells by an MHC-independent manner^[^^3^^]^. Kymriah and Yeskarta are two gene therapies based on CAR T-cell therapy, which has recently been approved by the US Food and Drug Administration. This fact illustrates the power of this therapy for the treatment of other cancers such as prostate cancer. In prostate cancer, PSMA is a valuable molecular marker for targeted therapy. PSMA is a type II integral membrane glycoprotein that is up-regulated during prostate cancer; therefore, it can serve as an attractive target for prostate cancer immunotherapy^[^^4^^]^.

Sera of camels and llamas have a special type of antibody without any light chains named heavy chain antibodies^[^^5^^]^. In the absence of the light chain, heavy chain antibodies bind antigens just by the variable domain of the heavy chains called nanobody or VHH^[^^6^^]^. The scFvs have two antigen-binding domains, but nanobodies have just one binding site; hence, nanobodies can be cloned easily in a multi-domain construct. In the previous preclinical and clinical studies, nanobodies have shown extremely low immunogenic potential^[^^7^^]^ due to the high sequence homology to human VH gene families^[^^8^^]^. Nanobodies can target antigens in the same manner as scFvs do. The above-mentioned characteristics make nanobodies as an ideal alternative instead of scFvs in CAR constructs. 

In this report, we designed and developed a second-generation CAR based on an anti-PSMA nanobody (NBPII-CAR). The CAR-T cells were functionally characterized in the Jurkat cells after co-culturing with the prostate cancer cells *in vitro*. This nanobody-based CAR was efficiently expressed on the transfected Jurkat cells and could specifically activate the Jurkat cells after recognizing PSMA on the prostate cancer cells. 

## MATERIALS AND METHODS

**Cell lines**

Jurkat E6.1 was purchased from Iranian Biological Resource Center (IBRC, Tehran, Iran) and human prostate cancer cell lines (LNCaP and DU-145) from the Pasteur Institute of Iran (Tehran, Iran). DU-145 cells were maintained in DMEM (Biosera, France), and the Jurkat and LNCaP cells in RPMI 1640 medium (Biosera) with 10% heat-inactivated fetal bovine serum (Biosera), 100 IU/ml of penicillin (Sigma-Aldrich, USA), and 100 µg/ml of streptomycin (Sigma-Aldrich). Cell lines were cultured at 37 °C with 95% humidity and 5% CO_2 _for 4-7 days before use.

**NBPII-CAR construct**

**Fig. 1:**
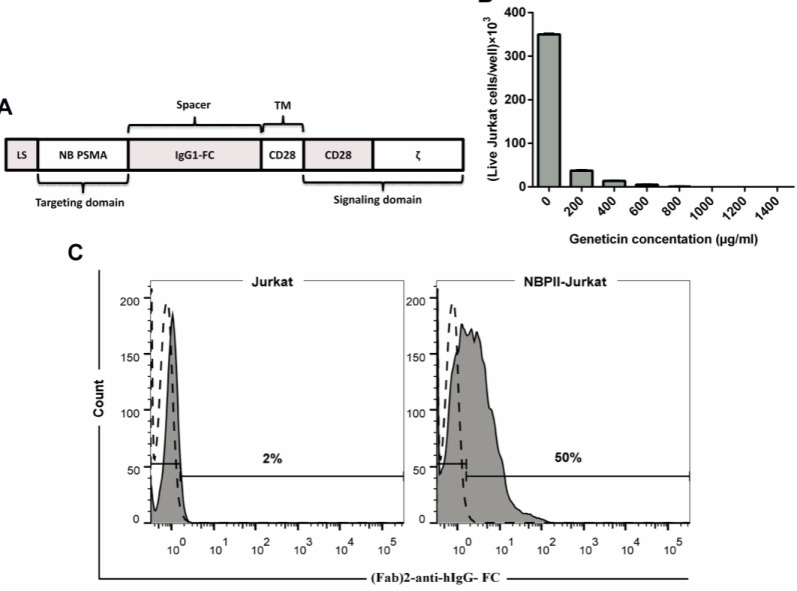
Schematic presentation of NBPII-CAR, kill curve for geneticin in Jurkat cells, and NBPII-CAR expression on Jurkat cells. (A) Schematic diagram of NBPII-CAR. The NBPII-CAR consists of a LS, anti-PSMA nanobody (NB PSMA), IgG1-FC domain (spacer), transmembrane domain of CD28 (TM), intracellular domain of CD28 (CD28), and signaling domain from ζ. (B) Histogram showing the viability of Jurkat cells in different antibiotic concentrations. As observed in the Figure, 1000 µg/ml is the minimum concentration of geneticin, which killed Jurkat cells after seven days. (C) NBPII-CAR expression was detected by flow cytometry. The filled histograms indicate NBPII-CAR-specific staining; the open and dashed line histograms indicate staining with isotype control antibody. Also, 50% of T cells expressed NBPII-CAR

A single-domain antibody fragment (nanobody) against PSMA (NBP) was used for target recognition (kindly provided by Dr. W.M. van Weerden) in the CAR construct^[^^9^^]^. Intracellular activation domains of human CD28 and activating components of ζ were linked to the transmembrane domain of CD28, and this complex was connected to nanobody via a spacer (IgG1-hinge CH2-CH3) to achieve flexibility in the extracellular region. This construct is a second-generation CAR, so called NBPII-CAR (Fig. 1A). NBPII-CAR was synthesized by Biomatik Company (Cambridge, Canada) and subcloned into the mammalian expression vector pcDNA3.1 (Invitrogen, CA, USA) named pNBPII-CAR. After confirmation with colony PCR, restriction enzyme mapping, and sequencing, the plasmid was propagated in *Escherichia coli* (DH5a).

**Electroporation**

Jurkat cells were electroporated with pNBPII-CAR by using the gene pulser electroporator (Bio-Rad, Munich, Germany). Briefly, Jurkat cells (6 10^6^) were mixed with 400 µl of FBS-free Opti-MEM medium (Invitrogen) and 20 µg of pNBPII-CAR. The cell suspension was incubated at room temperature for 15 minutes, then transferred into the electroporation cuvette with a 4-mm gap width and finally electroporated using Bio-Rad apparatus (set at 320 V, 950 µF). After 10 minutes on ice, the cells were transferred to four wells of a 24-well plate. Seventy-two hours after electroporation, NBPII-CAR expressing Jurkat cells were enriched by geneticin selection.

**Geneticin kill curve**

The Jurkat cells were exposed to increasing amounts of geneticin to determine the minimum geneticin concentration required to kill all Jurkat cells in a week period. The Jurkat cells (1 × 10^5^) were added to the wells of a 24-well tissue culture plate and after 16 hours, increasing concentrations of geneticin (0, 200, 400, 600, 800, 1000, 1200, and 1400 µg/ml) were added to the duplicated wells in complete RPMI-1640 medium and replaced after every two days. After seven days, Jurkat cells were counted, and the lowest concentration of geneticin that killed all Jurkat cells was determined.

**Detection of NBPII-CAR by flow cytometry**

After introducing NBPII-CAR into the Jurkat cells and enriching by geneticin, the surface expression of construct on the transfected cells was analyzed by flow cytometry. Cells (3 10^5^) were washed with PBS and re-suspended in 100 µl staining buffer (PBS containing 2% FBS with one µg per test FITC-conjugated goat anti-human IgG, Fcγ fragment-specific F(ab')2 (polyclonal, Invitrogen, Waltham, MA, USA) and incubated at 4 °C for 45 minutes. Jurkat cells were washed twice with PBS and then re-suspended in PBS and analyzed by flow cytometry.

**Activation of engineered Jurkat cells**

IL-2 secretion and CD25 expression were analyzed as T-cell activation markers. Prostate cancer cells were seeded in a 96-well tissue culture plate (2 10^4^/well). After an overnight culture, NBPII-CAR Jurkat cells were co-incubated with target cells E:T of 1:1 (2 10^4^/well) and 3:1 (6 10^4^/well). After 24 hours, the supernatant was collected for IL-2 assay using an ELISA kit (Quantikine Kit, R&D systems, Minneapolis, MN, USA). Moreover, effector cells were harvested, washed and re-suspended in 100 µl staining buffer containing 0.5 µg per test FITC-conjugated anti-human CD25 antibody (BC96 clone, BioLegend, San Diego, CA, USA) or 0.5 µg per test FITC-conjugated mouse IgG1, κ isotype control antibody (MOPC-21 clone, BioLegend). Jurkat cells were incubated at 4 °C for 45 minutes, then washed and re-suspended in PBS and analyzed by flow cytometry.

**Proliferation assay**

Proliferation of engineered Jurkat cells against prostate cancer cells was monitored by an XTT-based colorimetric assay, based on Jost *et al.*^[^^10^^]^. Reduction of XTT to formazan by viable tumor cells was monitored colorimetrically. Briefly, 48 hours after co-incubation of engineered and mock Jurkat cells with target cells (E:T of 1:1 and 3:1), XTT (1 mg/ml; Cell Proliferation Kit II; Roche Diagnostics, GmbH, Mannheim, Germany) was added, and the cells were incubated at 37 °C for 60 min. Reduction of XTT to formazan by viable cells was monitored colorimetrically at 450 nm. The following formula was used to calculate Jurkat cell proliferation rate. Finally, the proliferation rates of effector cells on LNCaP cells were normalized with the proliferation rate of effector cells on DU-145 cells. 

$\mathrm{Proliferation rate}\left(\%\right)=$

$\frac{\mathrm{OD}\left(\mathrm{experimental well}-\mathrm{tumor cells without effector}\right)}{\mathrm{OD}\left(\mathrm{corresponding number of effector cells}-\mathrm{media}\right)}\times 100$

**Statistical analysis**

Statistical analyses were performed using Prism software (GraphPad 6). Paired student’s *t*-test was used with a confidence interval of 95%.* p *values less than 0.05 were considered statistically significant.

## RESULTS

**NBPII-CAR expression on electroporated Jurkat cells**

Because of the low expression of NBPII-CAR after electroporation, we had to enrich engineered cells. For this reason, after transfection, geneticin was used as a selection marker to enrich NBPII-CAR-transfected cells. Kill curve assay was used to optimize the concentration of geneticin to kill all Jurkat cells. As Figure 1B shows, the minimum geneticin concentration, which killed all Jurkat cells, was 1000 µg/ml. Flow cytometry was employed to assess NBPII-CAR expression in transfected Jurkat cells. Ten days after electroporation and enriching with geneticin, the cells were analyzed for CAR expression. As indicated in Figure 1C, 50% of electroporated Jurkat cells expressed NBPII-CAR.

**IL-2 secretion following NBPII-CAR Jurkat cells activation**

**Fig. 2:**
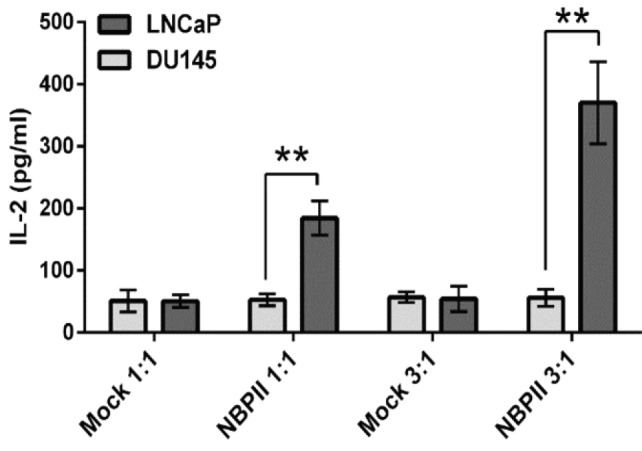
**Specific activation of engineered Jurkat cells after encountering PSMA** **-** **expressing cells. After 24 hours of co-culturing Jurkat cells with prostate cancer cells at E:T** **of** **1:1 and 3:1, IL-2 secretion by NBPII-CAR Jurkat cells was quantified by ELISA assay.** ^**^ ***p*** **<** **0.01 vs. DU-145 cells**

IL-2 secretion was monitored as an activation marker after the exposure of engineered Jurkat cells to prostate cancer cells. For this reason, Jurkat cells were co-cultured with LNCaP (PSMA^+^) and DU-145 (PSMA^-^) cells in E:T of 1:1 and 3:1 for 24 hours. Based on Figure 2, engineered cells secreted 180 pg/ml IL-2 in E:T of 1:1 and 370 pg/ml in E:T of 3:1. Both ratios were statistically significant as compared with those of DU-145 cells. Mock cells (electroporated with pCDNA3.1) did not show any significant IL-2 production upon co-culturing with LNCaP and DU-145 cells.

**CD25 expression on the surface of NBPII-CAR Jurkat cells**

**Fig. 3:**
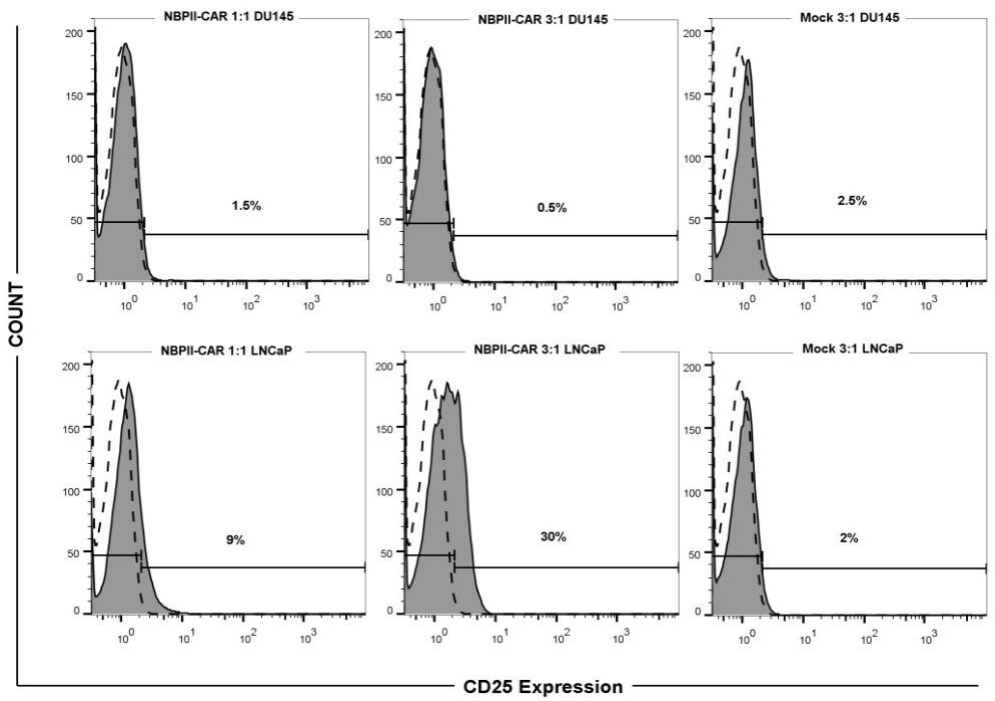
**Up-regulation of CD25 after T** **-** **cell activation. NBPII-CAR Jurkat cells and mock cells were co-cultured with prostate cancer cells at E:T (1:1 and 3:1). After 24 hours, harvested Jurkat cells were stained with PE-conjugated anti-CD25 antibody or isotype control antibody and analyzed by flow cytometry. The filled histograms indicate CD25-specific staining** **. T** **he open and dashed line histograms indicate staining with isotype control antibody. NBPII-CAR Jurkat cells are specifically activated by the target cells**

To determine if the NBPII-CAR could activate Jurkat cells after encountering PSMA, electroporated Jurkat cells were co-cultured with LNCaP and DU-145 cells in E:T of 1:1 and 3:1, respectively. Twenty-four hours after co-incubation, CD25 expression was analyzed as a common marker to assess T-cell activation. As Figure 3 shows, CD25 expression has increased in NBPII-CAR Jurkat cells up to 30% in 3:1 ratio when encountered LNCaP cells, while in Jurkat cells co-cultured with DU145 cells and mock cells, no alteration was detected.

**Proliferation of engineered Jurkat cells upon recognizing PSMA**

**Fig. 4:**
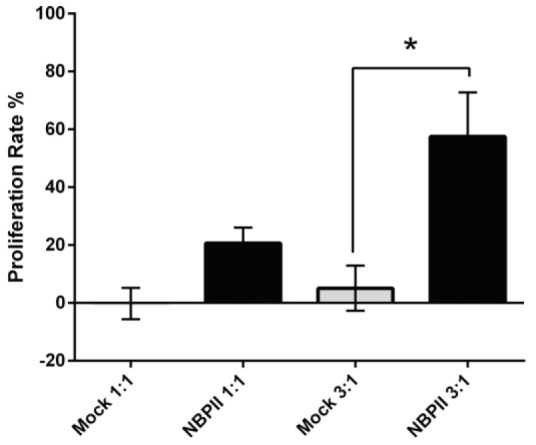
Proliferation of engineered Jurkat cells upon encountering PSMA-expressing cells. NBPII-CAR Jurkat cells and mock cells were co-cultured with prostate cancer cells in E:T of 1:1 and 3:1. After 48 hours, the proliferation rate of NBPII-CAR Jurkat cells was determined by XTT viability assay (^*^p < 0.05)

Proliferation rate of NBPII-CAR Jurkat cells after encountering prostate cancer cells was monitored. As demonstrated in Figure 4, the proliferation rate of NBPII-CAR Jurkat cells in E:T of 3:1 significantly increased to 60% when co-cultured with LNCaP cells relative to Jurkat cells encountered DU-145 cells.

## DISCUSSION

For the first time, we successfully constructed a second-generation CAR based on VHH against PSMA^[^^9^^]^. In this study, PSMA was chosen for targeting because it is strongly up-regulated in prostate cancer lesions. Some studies have reported that the expression of PSMA further enhances in high-grade, metastatic, and castration-resistant prostate cancer. Although the expression of this molecule is documented in the normal prostate epithelium, a subset of proximal renal tubules, small intestine, and salivary glands, the expression in these organs is 100–1000 folds less than in prostate cancer^[^^11^^,^^12^^]^. 

In our construct, nanobody against PSMA has been used as a prostate-targeting moiety. The classical scFvs with the mouse origin provoked human anti-mouse antibody response^[^^13^^]^, which can lead to the loss of efficacy of CAR T cells during treatment. Actually, the murine scFv may be more immunogenic than the natural antibody owing to the artificial linker between light and heavy chains. Such human anti-mouse antibodies were observed in the sera of patients who had been treated with CAR T cells derived from murine antibodies^[^^14^^]^. Repeated CAR T-cell administration led to anaphylaxis in one patient^[^^13^^]^ and limited the persistence of engineered T cells in the peripheral blood by anti-CAR antibodies^[^^15^^]^. However, Harding *et al.*^[^^16^^]^ reported that even a fully humanized monoclonal antibody could not prevent the production of anti-idiotypic antibodies. To overcome this limitation, we used nanobody instead of scFv in the CAR construct. Nanobodies confer many advantages over scFvs. First, their low immunogenic potential diminishes the risk of unwanted side effects and delays the clearance of nanobody-targeted CARs^[^^8^^]^. Second, their monomeric nature and small size make them ideal molecules for building multi-domain constructs^[^^17^^]^. Third, some scFv molecules have self-aggregative capacity, which may result in spontaneous CAR signaling and high background T-cell activation in the absence of the cognate target. Unlike scFvs, nanobodies have reduced aggregation tendency and are stable in harsh conditions^[^^18^^,^^19^^]^.

In this study, we combined functional domain of CD28, as a co-stimulatory domain. Because of deficiencies that were present in the first generation of CARs such as low cytokine secretion, low life span, and lack of proliferation, it was imperative that our CAR was designed as a second-generation CAR^[^^3^^,^^20^^]^. Optimal lymphocyte activation needs two signals, signal 1, related to ζ, and signal 2, related to co-stimulatory domain. Provision of ζ without CD28 signaling, the best characterized co-stimulatory domain, can result in a very low T-cell proliferation or induction of anergy and apoptosis^[^^21^^-^^23^^]^. It should be noted that both FDA approved CAR T cells have one co-stimulatory domain.

In the present study, we used electroporation, as a non-viral gene delivery system. Electroporation is a fast, safe and economical method to introduce DNA into the T cells^[^^24^^,^^25^^]^. By using this protocol, functional CAR-T cells were produced in less than two weeks. In addition, this method can be applied in combination with other genetic manipulation systems to engineer T cells. The introduction of DNA into the T cells is one of the crucial steps in CAR T-cell therapy. There are two conventional delivering techniques, including gamma-retroviral/lentiviral systems and electroporation. Although retroviral/lentiviral systems are widely applied, viral vectors can randomly integrate into the host genome and result in the latent hazard^[^^26^^]^. In addition, the preparation of virus particles is time-consuming and expensive and needs large technical requirements. 

After molecular designing and codon optimizing, NBPII-CAR was synthesized commercially and delivered in a cloning vector. The construct was then sub-cloned in a mammalian expression vector, pcDNA3.1(+), and the cloning procedure was validated by colony PCR, digestion, and DNA sequencing of the final construct . Since Jurkat cells are commonly used as a T-lymphocyte model to study T-cell activation events during preliminary testing of new CARs^[^^27^^-^^29^^]^, this cell line was chosen for the confirmation of NBPII-CAR surface expression and primary experiments. The expression of NBPII-CAR was monitored in transfected cells three days after electroporation. Geneticin was used as a selective antibiotic for increasing the expression of NBPII-CAR by Jurkat cells, and the percentage of NBPII-CAR Jurkat cells rose to the acceptable level after enriching by geneticin, according to Jensen *et al.*^[^^24^^]^ in 2000 and Brown *et al.*^[^^25^^]^ in 2015 (Fig. 1C). The majority of *in vitro* studies on prostate cancer are based on a limited number of cell lines: LNCaP, PC3, and DU145^[^^30^^-^^32^^]^. Therefore, the engineered Jurkat cells were co-cultured with LNCaP and DU145 cell lines, and then the activity of Jurkat cells was assessed. For checking the function and activity of NBPII-CAR in Jurkat cells, engineered Jurkat cells were co-incubated with prostate cancer cells in different ratios and IL-2 production, CD25 expression and proliferation were assessed.

The amount of IL-2 production was monitored by ELISA (Fig. 2) that showed elevated IL-2 secretion in LNCaP cells compared to DU-145 cells. The use of mock-transfected cells revealed no difference between positive and negative cells. Similar results were also found in the previous investigations^[^^33^^-^^35^^]^ in which nanobody-based CAR was constructed, and when engineered T cells faced the target cells, T cells were activated via nanobody-based CAR. The expression of CD25 (as an activation marker) on engineered Jurkat cells was analyzed by flow cytometry. After encountering with LNCaP cells, the expression rate of CD25 on NBPII-CAR Jurkat cells (Fig. 3) increased similar to the trend of IL-2 production. The CD25 expression was in line with those of Wing *et al.*^[^^36^^]^ that reported CAR T cells targeting the folate receptor alpha successfully infiltrated pre-established xenograft tumors and increased CD25 expression as an activation marker on T cells^[^^36^^]^. Subsequently, we assessed proliferation capacity of NBPII-CAR Jurkat cells. NBPII-CAR Jurkat cells proliferated more than the mock-electroporated Jurkat cells (Fig. 4) that support data obtained by Maher and collegous^[^^20^^]^. They showed that in the physiological condition, the optimal T-cell activation depends on the engagement of one or more co-stimulatory receptor. Lack of CD28 domain in construct can result in very low T-cell proliferative response^[^^20^^]^. These results provide a basis for further analysis of NBPII-CAR in peripheral blood mononuclear cells and *in vivo* modeling and also for future clinical studies.

Here, we described the ability of CAR-bearing T cells to recognize PSMA on the prostate cancer cell line that leads to the activation of T cells. These results clearly show the possibility of using nanobody-based CAR T cells for targeted immunotherapy. This new anti-PSMA CAR might be used as a promising candidate for clinical applications in the prostate cancer therapy.
